# Recurrent visceral leishmaniasis relapses in HIV co-infected patients are characterized by less efficient immune responses and higher parasite load

**DOI:** 10.1016/j.isci.2022.105867

**Published:** 2022-12-23

**Authors:** Yegnasew Takele, Tadele Mulaw, Emebet Adem, Rebecca Womersley, Myrsini Kaforou, Susanne Ursula Franssen, Michael Levin, Graham Philip Taylor, Ingrid Müller, James Anthony Cotton, Pascale Kropf

**Affiliations:** 1 Department of Infectious Disease, Imperial College London, London, UK; 2 Leishmaniasis Research and Treatment Centre, University of Gondar, Gondar, Ethiopia; 3 Wellcome Sanger Institute, Wellcome Genome Campus, Hinxton, UK

**Keywords:** Virology, Immunology, Immune response

## Abstract

Visceral leishmaniasis (VL) and HIV co-infection (VL/HIV) has emerged as a significant public health problem in Ethiopia, with up to 30% of patients with VL co-infected with HIV. These patients suffer from recurrent VL relapses and increased mortality. Those with a previous history of VL relapses (recurrent VL/HIV) experience increased VL relapses as compared to patients with HIV presenting with their first episode of VL (primary VL/HIV). Our aim was to identify drivers that account for the higher rate of VL relapses in patients with recurrent VL/HIV (n = 28) as compared to primary VL/HIV (n = 21). Our results show that the relapse-free survival in patients with recurrent VL/HIV was shorter, that they had higher parasite load, lower weight gain, and lower recovery of all blood cell lineages. Their poorer prognosis was characterized by lower production of IFN-gamma, lower CD4^+^ T cell counts, and higher expression of programmed cell death protein 1 (PD1) on T cells.

## Highlights

• VL/HIV co-infection is a significant public health problem in Ethiopia
• Patients with VL/HIV suffer from recurrent VL relapses and increased mortality
• Patients with recurrent VL/HIV relapse more often than patients with primary VL/HIV
• Patients with recurrent VL/HIV have a less efficient immune response

## Graphical abstract

**Figure:**
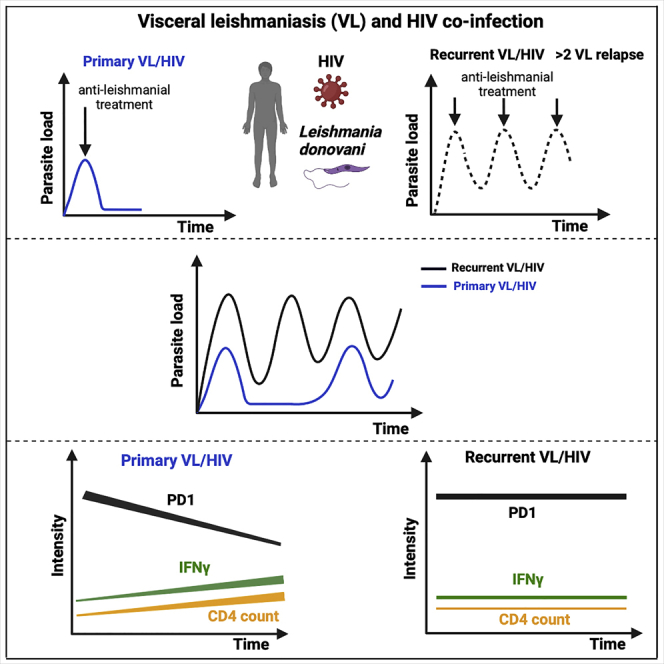

## Introduction

Visceral leishmaniasis (VL), also named kala-azar, is a potentially fatal neglected tropical disease, caused by parasites of the genus *Leishmania*. An estimated 50,000 to 90,000 new cases of VL occur worldwide annually, but only 17,082 new cases of VL were reported in 2018, with Brazil, Ethiopia, India, South Sudan, and Sudan, each reported >1000 VL cases, together representing 83% of all cases globally.^1^ Because of the remote location of VL endemic areas and the lack of surveillance, it is widely accepted that this is a vast underestimation of the real burden of VL in endemic areas. VL imposes a huge pressure on the developing countries and delays economic growth, with an approximate annual loss of 2.3 million disability-adjusted life years.^2^

In Ethiopia, where this study took place, VL is one of the most significant vector-borne diseases: over 3.2 million people are at risk of infection.^3^ VL is caused by infections with parasites of the *Leishmania* (*L.*) *donovani* species complex. Not all infected individuals will develop the disease: some will stay asymptomatic, but in those who develop VL, the disease is characterized by hepatosplenomegaly, fever, pancytopenia, and severe weight loss; this stage of the disease is mostly fatal if left untreated.^4^^,^^5^^,^^6^ Following the HIV-1 pandemic, VL has emerged as an opportunistic infection: HIV infection increases the risk of developing symptomatic VL and VL accelerates the progression of HIV infection to AIDS.^7^^,^^8^ HIV co-infection presents a significant challenge in the prevention and control of VL^9^^,^^10^: VL/HIV co-infected patients experience increased rates of VL relapse, mortality, and treatment failure compared to patients with VL alone.^5^^,^^6^^,^^11^^,^^12^^,^^13^^,^^14^ Our knowledge of the immunology of VL/HIV co-infections is still limited. Increased levels of soluble CD40L have been shown to be associated with the resolution of VL.^15^ In a separate study, it was shown that decreased levels of CD40L and increased levels of neopterin, a molecule associated with activation of macrophages, were present in the plasma of HIV/VL as compared to patients with HIV.^16^ Van den Bergh et al. suggested that myeloid-derived suppressor cells might play a role in severely immunocompromised HIV/VL co-infected patients.^17^ We have shown previously that the activity of arginase, an enzyme with immunoregulatory properties, was significantly higher in the blood of VL/HIV co-infected patients as compared to patients with VL, indicating that increased arginase activity contributes to T cell hyporesponsiveness in VL/HIV co-infected patients.^18^ A recent study has identified a 4-gene immune signature that could identify patients who fail treatment from those who are successfully treated.^19^ Santos-Oliveira et al. showed that increased numbers of activated T lymphocytes are present in VL/HIV co-infected patients, even in those with undetectable viral load.^20^ This increase in T cell activation was associated with lipopolysaccharide levels.^21^ Low CD4^+^ T cell counts are also a hallmark of patients with VL/HIV.^5^^,^^14^^,^^22^

We have recently shown that throughout follow-up, as compared to patients with VL, patients with VL/HIV display:^11^

- higher parasite load;
- impaired antigen-specific IFNγ production by whole blood cells;
- lower CD4^+^ T cell counts;
- higher PD1 expression on CD4^+^ and CD8^+^ T cells.

Our data also show that over a period of three years, 78.1% of patients with VL/HIV experience at least one relapse.^11^ The following markers have been shown to predict VL relapse in patients with VL/HIV:

- failure to clear parasite load after anti-leishmanial treatment,^14^ as well as presence of circulating *Leishmania* DNA;^23^
- low CD4^+^ T cell counts;^5^^,^^14^
- previous VL episodes in patients with VL/HIV;^5^^,^^12^^,^^14^ however, a study with patients with VL/HIV in Ethiopia showed no association between a previous history of relapse and increased risks of future relapse.^24^

In a study where patients with VL/HIV received VL prophylaxis treatment after initial cure of VL, it was shown that the group of patients who did not relapse displayed lower soluble CD14 and anti-*Leishmania* IgG3 levels, as well as less activated T cells, suggesting that these patients could control immune activation more efficiently.^25^ Recently, a study assessed the involvement of the thymus in the replenishment of T cells: their results suggest that in VL/HIV co-infected patients who do not relapse, more new emigrant T cells can be detected that might contribute to the control of parasite replication.^26^ The work by Casado et al. also showed that patients with VL/HIV had an even worse immunological status as compared to ART-treated patients with HIV who failed to improve their CD4^+^ T cell counts.^27^

In our recent study, we showed that patients with VL/HIV who did not relapse after initial clinical cure had lower parasite loads as measured by RNAseq in whole blood, produced higher levels of antigen-specific IFNγ, maintained higher CD4^+^ and CD8^+^ T cell counts, and lower PD1 expression on CD4^+^ and CD8^+^ T cells, as compared to patients with VL/HIV who did relapse.^11^ In this study,^11^ out of the 49 patients with HIV who presented with VL at the treatment center, 21 (43%) patients presented with their first episode of VL (patients with primary VL/HIV) and 28 (57%) had a previous history of VL (patients with recurrent VL/HIV). It has been previously shown that patients with recurrent VL/HIV experience increased VL relapses as compared to primary VL/HIV.^5^^,^^6^ However, the mechanisms resulting in this increased relapse rate are poorly characterized.

In the current study, we assessed the impact of a previous history of VL relapse on relapse-free survival, clinical parameters, and the immune response and compared those to patients with HIV presenting with their first episode of VL. To test this, we performed an extensive follow-up study of patients with primary and recurrent VL/HIV, from the time of VL diagnosis to 6–12 months post the end of treatment, determined the frequency of patients with VL/HIV who remain relapse-free over time, and identified clinical and immunological markers associated with VL relapse in these two cohorts.

## Results

### Higher relapse-free survival in patients with P VL/HIV

**Figure 1:**
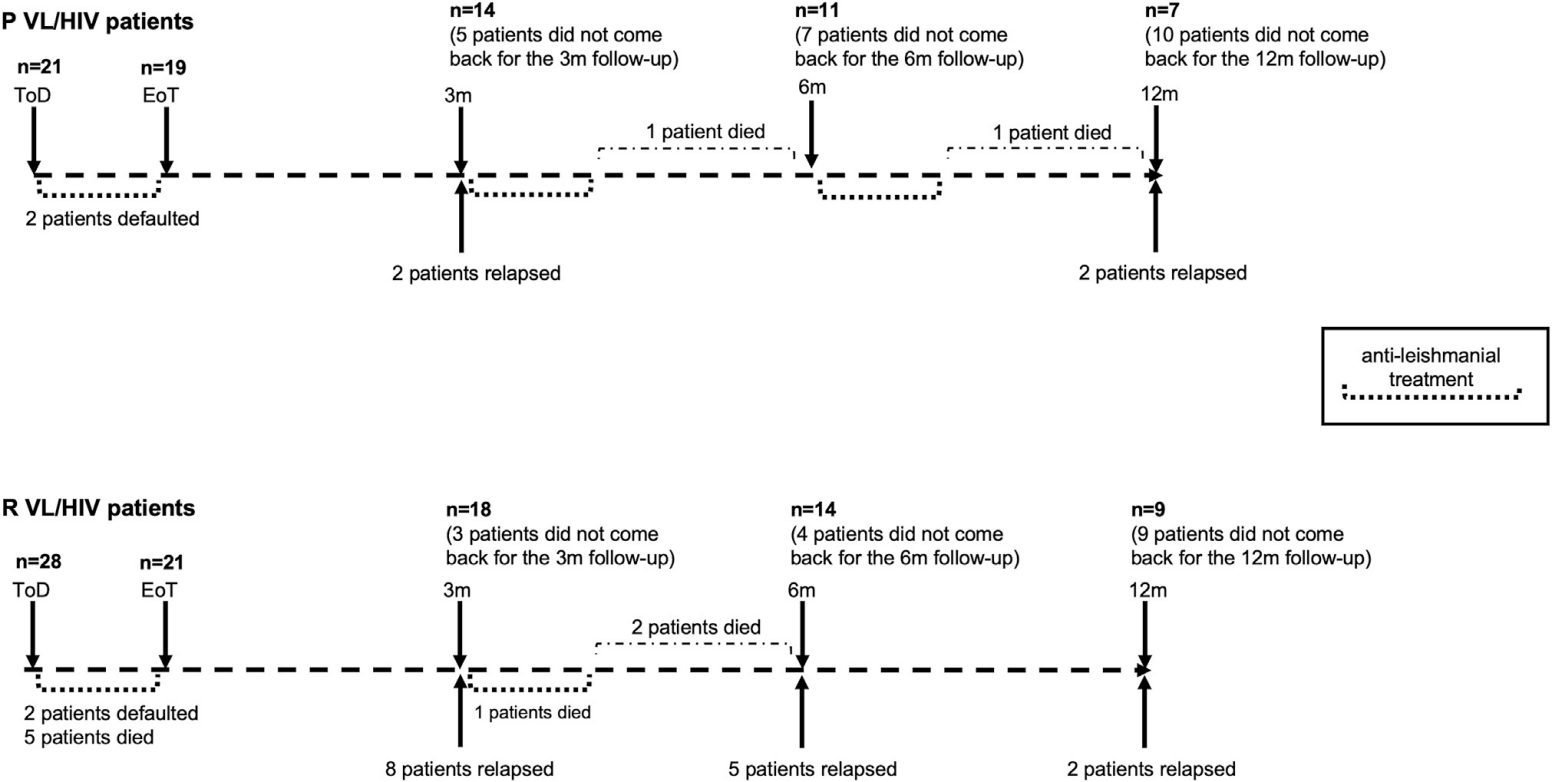
Follow-up of patients with primary and recurrent VL/HIV 21 patients with P and 28 patients with R VL/HIV were followed for up to 6–12m. ToD = Time of Diagnosis; EoT = End of Treatment; 3m = 3 months post EoT; 6–12m = 6–12 months post EoT.

**Figure 2:**
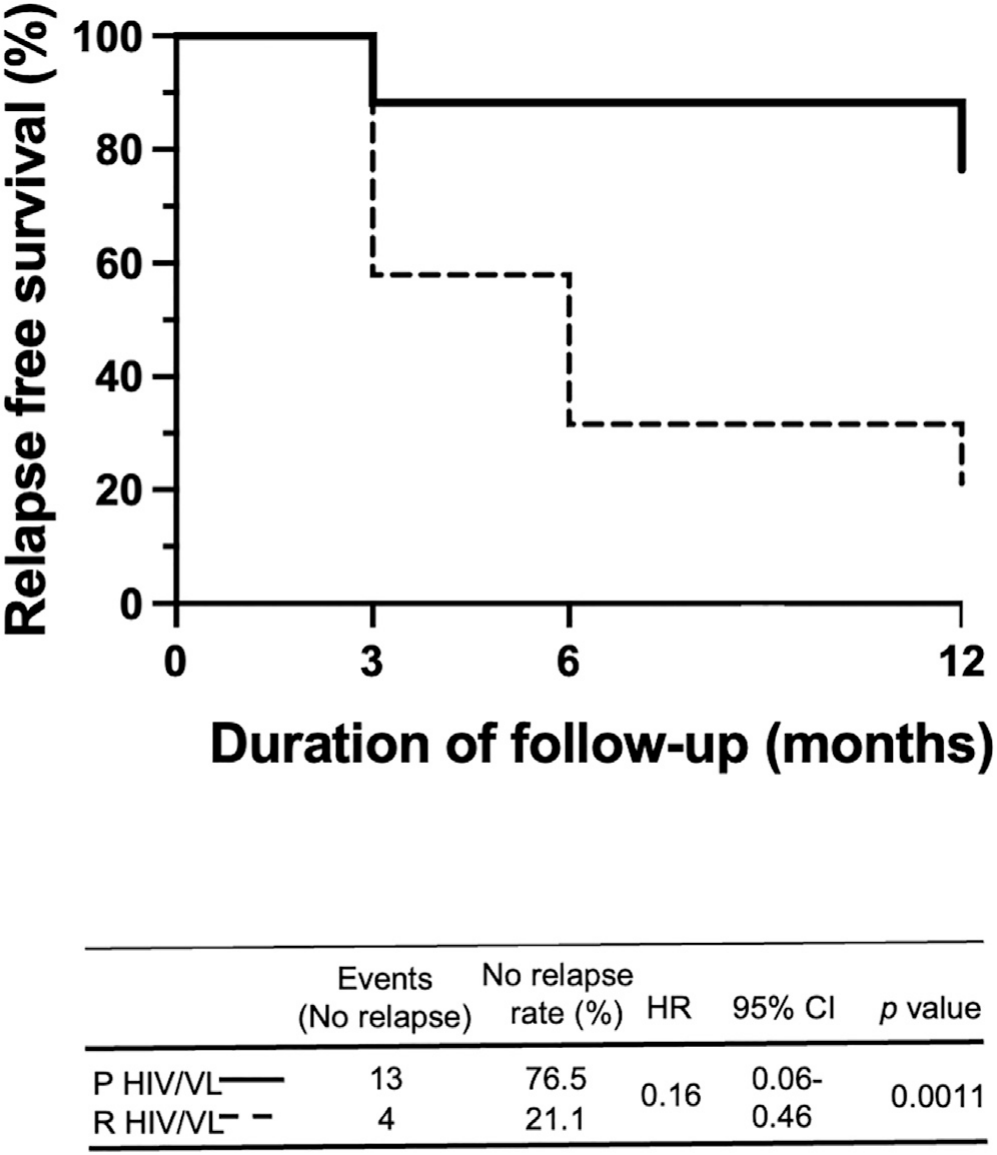
Relapse-free survival Kaplan-Meier curves of participant VL relapses comparing patients with P VL/HIV to R VL/HIV. The hazard ratios (with 95% confidence intervals and p values) obtained from the Cox model indicated the change in relapse-free survival following treatment for VL for these groups. HR, hazard ration; CI, confidence interval.

We previously reported that despite initial clinical cure, 78.1% of patients with VL/HIV relapsed at least once.^11^ In this cohort of patients with VL/HIV, 21 patients with HIV presented with their first episode of VL (patients with primary VL/HIV, P VL/HIV) and 28 presented with VL but had already experienced previous VL (recurrent VL/HIV, R VL/HIV) (Table 1, Figure 1). To assess if the absence of a previous history of VL results in a longer relapse-free survival, a better prognosis, and a stronger immune response, these two cohorts of patients were followed for a period of 6–12 months (Figure 1) and detailed clinical and immunological data were collected. As shown in Figure 2, the percentage of relapse-free survival over a period of 12 months was significantly higher in patients with P as compared to patients with R VL/HIV (76.5% vs 21.1%, respectively, p = 0.0011). More patients with R VL/HIV relapsed earlier as compared to patients with P VL/HIV: 42.1% vs 11.8% at 3 months, respectively.

**Table 1:** atients with recurrent VL/HIV: number of relapses at ToD

Number of relapses	Number of patients
1	4
2	7
3	5
4	2
5	1
6	1
7	1
9	2
10	2
12	1
14	2

Number of previous relapses in patients with R VL/HIV (n = 28) at ToD when this study was started. ToD = time of diagnosis.

### Increased parasite loads in patients with R VL/HIV

**Figure 3:**
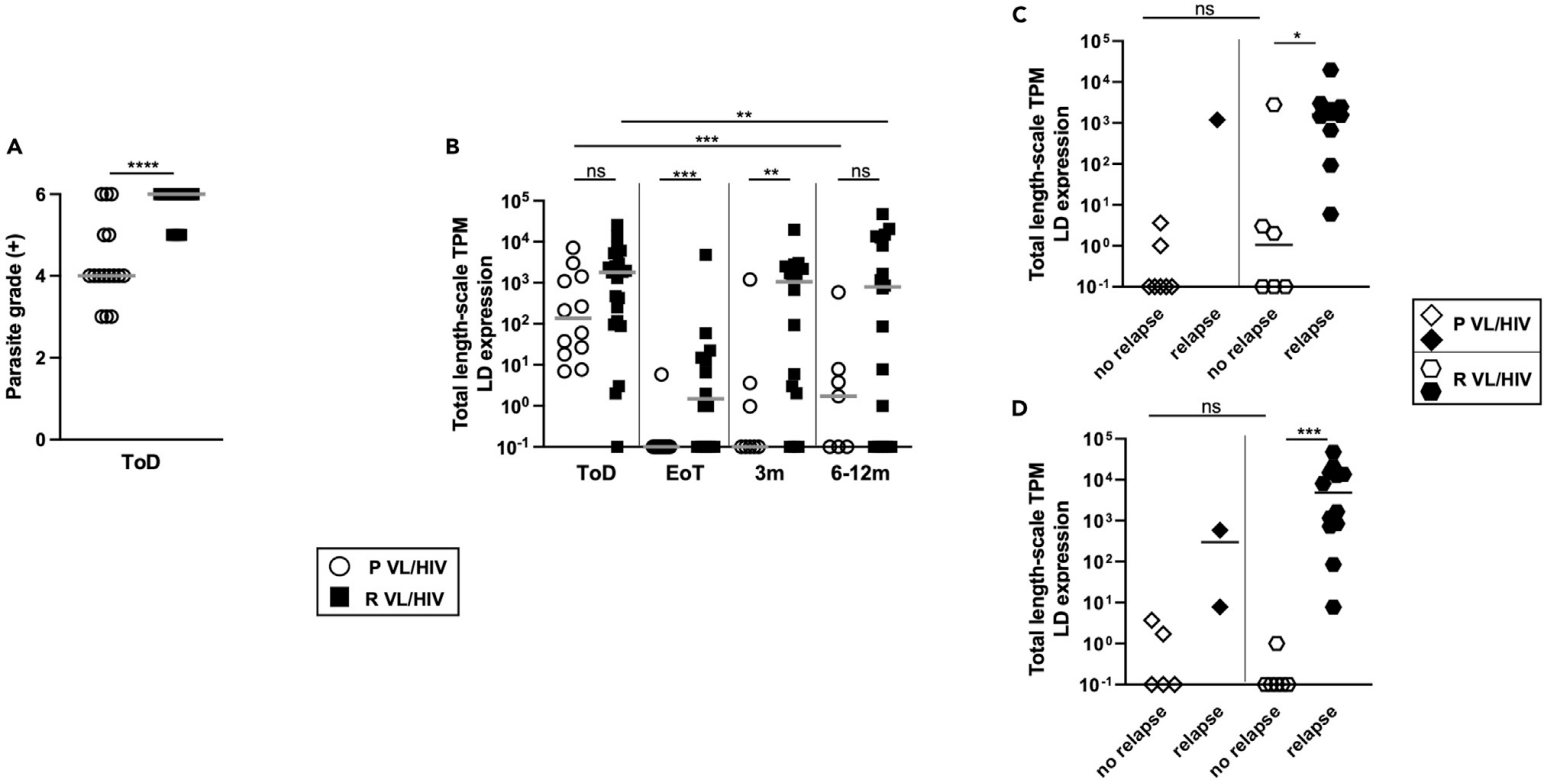
Parasite load (A) Quantification of *Leishmania* amastigotes in smears of splenic aspirates collected from patients with P VL/HIV (n = 16) and R VL/HIV (n = 26) at ToD. (B) Quantification of the total expression of *L. donovani* (LD) mRNA in blood from patients with P (ToD: n = 12, EoT: n = 16, 3m: n = 8, 6-12m: n = 7) and R VL/HIV (ToD: n = 23, EoT: n = 14, 3m: n = 16, 6-12m: n = 18). (C) Comparison of the total expression of LD mRNA in blood from patients with P VL/HIV who did not relapse (n = 7) and those who did relapse (n = 1) and R VL/HIV who did not relapse (n = 6) and those who did relapse (n = 10) 3m after successful anti-leishmanial treatment. (D) Comparison of the total expression of *L. donovani* mRNA in blood from P VL/HIV who did not relapse (n = 5) and those who did relapse (n = 2) and R VL/HIV who did not relapse (n = 6) and those who did relapse (n = 12) 6–12m after successful anti-leishmanial treatment. If a patient did not relapse during the 2 time points of follow-up and if a patient relapsed at both 3, 6–12 months, this is represented as 2 measurements. ns = not significant. Each symbol represents the value for one individual, the straight lines represent the median. Statistical differences between P and R VL/HIV patients (A) at each time point (B) were determined using a Mann-Whitney test; statistical differences between the 4 different time points (B) for each cohort of patients were determined by Kruskal-Wallis test.LD mRNA = *L. donovani* mRNA. ToD = Time of Diagnosis; EoT = End of Treatment; 3m = 3 months post EoT; 6-12m = 6–12 months post EoT. See also Table S1.

Next, we compared parasite grades in splenic aspirates of patients with P and R VL/HIV at time of diagnosis (ToD). As shown in Figure 3A, parasite grades were significantly lower in patients with P than R VL/HIV (p < 0.0001). Parasite grade can only be measured when the spleen is palpable and >3 cm below the costal margin; it is therefore mainly measured at ToD. We have previously shown that RNAseq can be used to measure the total expression of *L. donovani* mRNAs (*Ld* mRNA) in blood.^11^ The median *Ld* mRNA was lower at ToD in patients with P VL/HIV; this was not statistically significant (Table S1, Figure 3B). At the end of treatment (EoT) and at 3 months, there was significantly less *Ld* mRNA in P VL/HIV (Figure 3B, Table S1). At 6–12 months, despite a higher median *Ld* mRNA in patients with R HIV/VL, the differences between the 2 groups were not significant (Table S1, Figure 3B). As shown in Figure 2, significantly more patients with P VL/HIV remain relapse-free during follow-up (3 and 6–12 month time points). To assess whether these patients have a lower parasite load, we further divided each cohort of patients with P and R VL/HIV into 2 subgroups: those who did not relapse and those who did relapse after initial cure, at both 3 and 6–12 months. The median *Ld* mRNA levels were similar between patients with P and R VL/HIV who did not relapse during follow-up at both time points (Figures 3C and 3D). As expected, an increase in parasite load was observed in patients with P and R VL/HIV who relapsed (Figures 3C and 3D). However, we could only collect PAXgene tubes from one (3 months) and two (6–12 months) patients with P VL/HIV, so it is not possible to draw meaningful conclusions from these data.

Despite being on ART, the majority of patients with P VL/HIV and just under half of the R VL/HIV cohort had detectable plasma viral load (Table S2A). There were no significant differences in plasma viral load (Table S2B) between patients with P and R VL/HIV at each time point and over time. No significant differences in viral load were observed between patients with P and R VL/HIV who relapsed and those who did not relapse during follow-up (data not shown).

### Poorer weight gain and higher spleen sizes in patients with R VL/HIV

**Figure 4:**
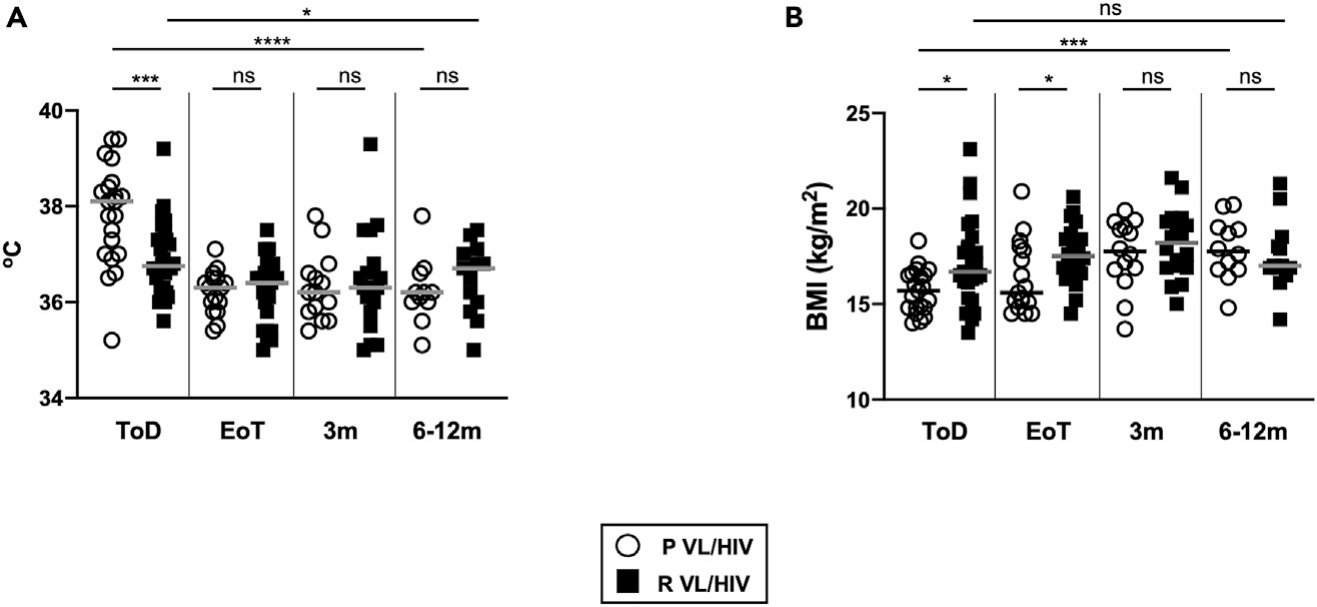
Clinical parameters (A) Body temperature was measured in patients with P VL/HIV (ToD: n = 21, EoT: n = 16, 3m: n = 14, 6-12m: n = 11) and R VL/HIV (ToD: n = 28, EoT: n = 22, 3m: n = 18, 6-12m: n = 13). (B) BMI was calculated for patients with P VL/HIV (ToD: n = 21, EoT: n = 17, 3m: n = 14, 6-12m: n = 12) and R VL/HIV (ToD: n = 28, EoT: n = 22, 3m: n = 18, 6-12m: n = 13). Each symbol represents the value for one individual, and the straight lines represent the median. Statistical differences between patients with P VL/HIV and R VL/HIV at each time point were determined using a Mann-Whitney test; statistical differences between the 4 different time points for each cohort of patients were determined by Kruskal-Wallis test. ToD = Time of Diagnosis; EoT = End of Treatment; 3m = 3 months post EoT; 6-12m = 6–12 months post EoT. ns = not significant.

Next, we assessed whether patients with P and R VL/HIV presented with different clinical manifestations. At ToD, fever was significantly higher in patients with P VL/HIV as compared to patients with R VL/HIV (Figure 4A) but normalized thereafter to levels similar to controls (controls: 36.0 ± 0.1°C, p > 0.05, data not shown). No significant differences in fever were observed between patients with P and R VL/HIV who relapsed and those who did not relapse during follow-up (data not shown).

The median BMI of each group of patients with VL/HIV patients was below the normal value of 18.5^28^ throughout follow-up and was significantly higher in patients with R than in patients with P VL/HIV at ToD and EoT (p = 0.0173 and p = 0.0192, respectively, Figure 4B). The BMI of patients with P VL/HIV, but not R VL/HIV, increased significantly over time (p = 0.0010). No significant differences in BMI were observed between patients with P and R VL/HIV who relapsed and those who did not relapse during follow-up (data not shown).

**Figure 5:**
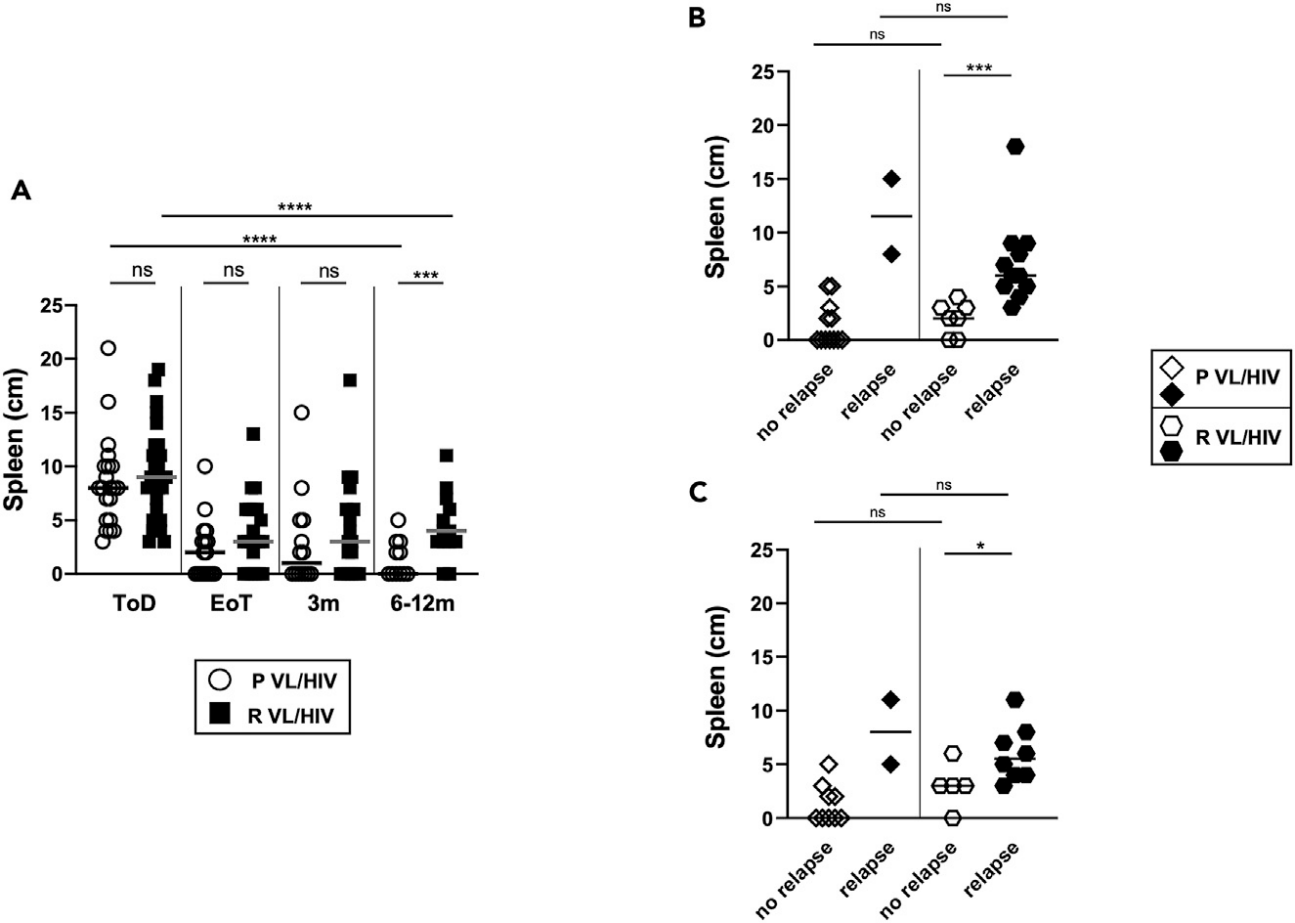
Spleen sizes (A) Spleen size as measured in cm below the costal margin on patients with P VL/HIV (ToD: n = 21, EoT: n = 17, 3m: n = 14, 6–12m: n = 11) and R VL/HIV (ToD: n = 28, EoT: n = 22, 3m: n = 18, 6–12m: n = 13). (B) Comparison of the spleen of patients with P VL/HIV who did not relapse (n = 12) and those who did relapse (n = 2) and patients with R VL/HIV who did not relapse (n = 7) and those who did relapse (n = 11) 3m after successful anti-leishmanial treatment. (C) Comparison of the spleen of patients with P VL/HIV who did not relapse (n = 9) and those who did relapse (n = 2) and patients with R VL/HIV who did not relapse (n = 5) and those who did relapse (n = 8) 6–12m after successful anti-leishmanial treatment. If a patient did not relapse during the 2 time points of follow-up and if a patient relapsed at both 3, 6–12 months, this is represented as 2 measurements. Each symbol represents the value for one individual, the straight lines represent the median. Statistical differences between 2 groups were determined using a Mann-Whitney test; statistical differences between the 4 different time points for each cohort of patients were determined by Kruskal-Wallis test.ToD = Time of Diagnosis; EoT = End of Treatment; 3m = 3 months post EoT; 6-12m = 6–12 months post EoT. ns = not significant.

Since hepatosplenomegaly is a typical sign of patients with VL/HIV, spleen and liver sizes as measured below the costal margin are systematically recorded when patients present to the clinic. As shown in Figure 5A, spleen sizes were similar in both groups at ToD, EoT, and 3 months and decreased throughout follow-up, but were significantly higher at 6–12 months in patients with R VL/HIV (p = 0.0061). To assess whether this increase in spleen size was due to VL relapse, we compared the spleen size of patients who did not relapse with those who relapsed after initial cure in P and R VL/HIV groups. As shown in Figures 5B and 5C, there was no significant difference in spleen size between the patients who did not relapse during follow-up in both cohorts (patients with P and R VL/HIV). As expected, the spleen sizes were higher in patients with P and R VL/HIV who relapsed during follow-up in both groups (Figures 5B and 5C). Of note, spleen sizes were measured in only two patients with P VL/HIV who relapsed at 3 months and 6–12 months; it is therefore not possible to draw meaningful conclusions from these data.

The liver was also clearly measurable below the costal margin at ToD in both groups of patients and decreased throughout follow-up (Table S3). No significant differences in liver sizes were observed between patients with P and R VL/HIV who relapsed and those who did not relapse during follow-up (data not shown).

### Lower recovery of all blood cell lineages in patients with R VL/HIV

White blood cell counts (WBCs) were similar in both groups of patients with VL/HIV at ToD and increased at EoT; however, at 6–12 months, patients with R VL/HIV had significantly lower WBCs than patients with P VL/HIV (Table S4A). Red blood cells (RBCs) increased significantly in patients with P VL/HIV until the 3 month time point and plateaued at 6–12 months; however, no significant improvement in RBCs was observed in the blood of patients with R VL/HIV throughout the follow-up (Table S4B). Similarly, platelet (PLT) counts increased at EoT in patients with P VL/HIV, plateaued thereafter and were higher than in patients with R VL/HIV at 3 and 6–12 months (Table S4C); PLT counts of patients with R VL/HIV did not change significantly throughout the follow-up (Table S4C). Despite an increase in WBC, RBC, and PLT counts in the blood of patients with P VL/HIV, these values still remained significantly lower than those of healthy controls (p < 0.0001, data not shown).

### Less efficient antigen-specific production of IFNγ, but not IL-10, by whole blood cells from patients with R VL/HIV

**Figure 6:**
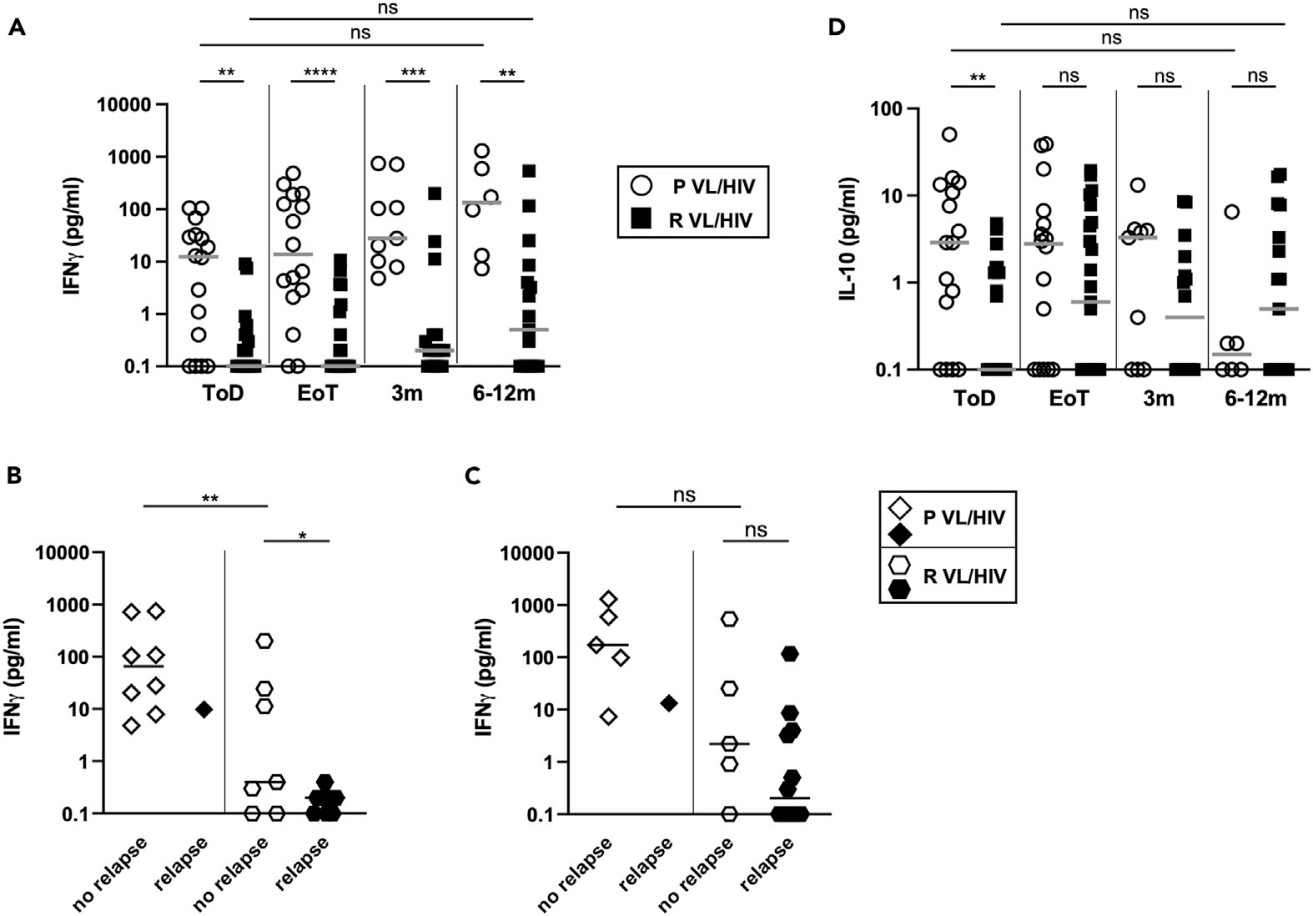
Whole blood assay: antigen-specific production of IFNγ and IL-10 Whole blood cells from patients with P VL/HIV (ToD: n = 16, EoT: n = 16, 3m: n = 9, 6-12m: n = 6) and R VL/HIV (ToD: n = 22, EoT: n = 23, 3m: n = 16, 6-12m: n = 17) were cultured in the presence of SLA and PBS. (A**)** IFNγ and (D**)**. IL-10 levels in the supernatant were measured by ELISA after 24 h and the values obtained with PBS alone was deducted from the value obtained with SLA. (B) Comparison of IFNγ levels in the supernatant of whole blood cells from patients with P VL/HIV who did not relapse (n = 8) and those who did relapse (n = 1) and patients with R VL/HIV who did not relapse (n = 7) and those who did relapse (n = 9) 3m after successful anti-leishmanial treatment. (C) Comparison of IFNγ levels in the supernatant of whole blood cells from patients with P VL/HIV who did not relapse (n = 5) and those who did relapse (n = 1) and patients with R VL/HIV who did not relapse (n = 5) and those who did relapse (n = 12) 6-12m after successful anti-leishmanial treatment. If a patient did not relapse during the 2 time points of follow-up and if a patient relapsed at both 3, 6–12 months, this is represented as 2 measurements. Statistical differences between two groups were determined using a Mann-Whitney test; statistical differences between the 4 different time points for each cohort of patients were determined by Kruskal-Wallis test.ToD = Time of Diagnosis; EoT = End of Treatment; 3m = 3 months post EoT; 6-12m = 6–12 months post EoT. ns = not significant. See also Table S5.

One of the hallmarks of patients with VL/HIV is the inefficiency of whole blood cells to produce IFNγ in response to *Leishmania*-specific stimulation at time of diagnosis and throughout follow-up.^11^ Here, we compared *Leishmania*-specific IFNγ production by whole blood cells from patients with P and R VL/HIV. Results presented in Figure 6A show that whereas the levels of IFNγ did not significantly change over time in both groups, whole blood cells from patients with R VL/HIV produced significantly less IFNγ than whole blood cells from patients with P VL/HIV throughout follow-up. Next, we assessed whether whole blood cells from patients with P and R VL/HIV who did not relapse after initial cure produced more IFNγ. As shown in Figures 6B and 6C, there was more *Leishmania*-specific IFNγ produced by whole blood cells from patients with P VL/HIV who did not relapse as compared to patients with R VL/HIV who did not relapse at 3 months, but not at 6–12 months. In the R VL/HIV group, the levels of IFNγ were significantly higher in patients who had not relapsed at 3 months, but not at 6–12 months (Figures 6B and 6C). Of note, blood was collected from one patient with P VL/HIV who relapsed during follow-up at 3 months and at 6–12 months; it is therefore not possible to draw meaningful conclusions from these data.

The production of IFNγ in response to PHA remained similar in both groups throughout follow-up (Table S5A).

We have previously shown that *Leishmania*-specific production of IL-10 was not associated with disease severity.^11^ Our results presented in Figure 6D show that as compared to patients with P VL/HIV, antigen-specific production of IL-10 by R VL/HIV whole blood cells was significantly lower at ToD but similar at EoT, 3 and 6–12 months. No significant differences in antigen-specific IL-10 were observed between patients with P and R VL/HIV who relapsed and those who did not relapse during follow-up (data not shown).

The production of IL-10 in response to PHA increased significantly over time in both groups but was lower at 6–12 months in patients with R VL/HIV (Table S5B).

### Lower CD4^+^ T cell counts and higher PD1 expression in patients with R VL/HIV

**Figure 7:**
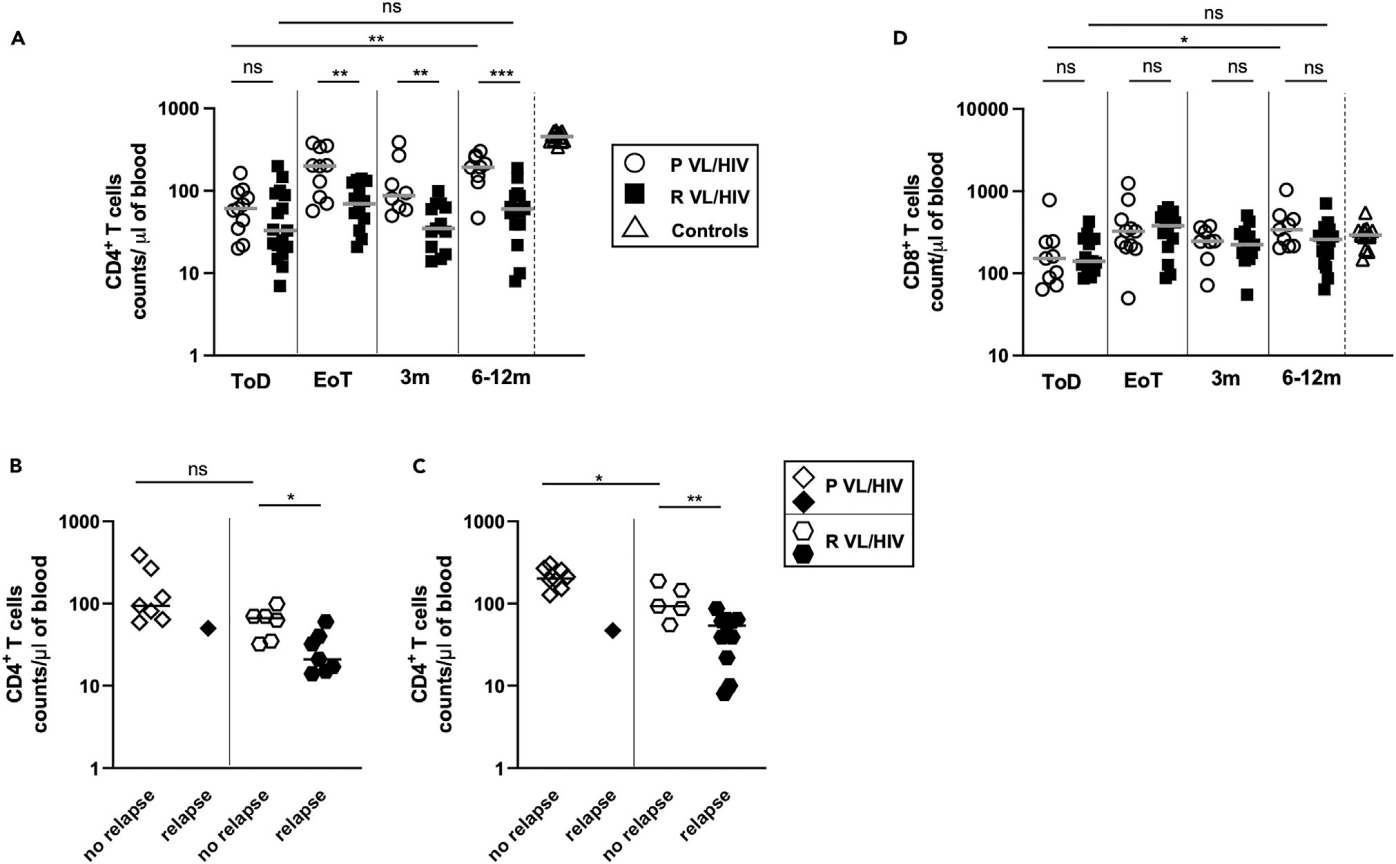
CD4^+^ and CD8^+^ T cell counts (A) CD4^+^ T cell counts in the blood of patients with P VL/HIV (ToD: n = 11, EoT: n = 10; 3m: n = 8, 6-12m: n = 9) and R VL/HIV (ToD: n = 17, EoT: n = 14, 3m: n = 13, 6-12m: n = 16). (B) Comparison of CD4^+^ T cell counts in the blood of patients with P VL/HIV who did not relapse (n = 7) and those who did relapse (n = 1) and patients with R VL/HIV who did not relapse (n = 6) and those who did relapse (n = 7) 3m after successful anti-leishmanial treatment. (C) Comparison of CD4^+^ T cell counts in the blood of patients with P VL/HIV who did not relapse (n = 8) and those who did relapse (n = 1) and patients with R VL/HIV who did not relapse (n = 5) and those who did relapse (n = 11) during 6-12m after successful anti-leishmanial treatment. (D) CD8^+^ T cell counts in the blood of patients with P VL/HIV (ToD: n = 13, EoT: n = 12, 3m: n = 8, 6-12m: n = 6) and R VL/HIV (ToD: n = 11, EoT: n = 12, 3m: n = 13, 6-12m: n = 10) were measured by flow cytometry. If a patient did not relapse during the 2 time points of follow-up and if a patient relapsed at both 3, 6–12 months, this is represented as 2 measurements. Statistical differences between two groups were determined using a Mann-Whitney test; statistical differences between the 4 different time points for each cohort of patients were determined by Kruskal-Wallis test.ToD = Time of Diagnosis; EoT = End of Treatment; 3m = 3 months post EoT; 6-12m = 6–12 months post EoT. ns = not significant.

Our previous results showed that in patients with VL/HIV, the failure to restore antigen-specific production of IFNγ correlated with persistently low CD4^+^ T cell counts and high expression of PD1 on CD4^+^ T cells.^11^ Results presented in Figure 7A show that despite low CD4^+^ T cell counts in both patients with P and R VL/HIV throughout follow-up as compared to controls (p < 0.0001), CD4^+^ T cell counts increased significantly in the P VL/HIV group, but not in the R VL/HIV group (Figure 7A). To assess whether patients with P and R VL/HIV who did not relapse after clinical cure had higher CD4^+^ T cell counts, the 2 cohorts were subdivided into patients who relapsed and those who did not. Results presented in Figures 7B and 7C show that patients with P and R VL/HIV who did not relapse had similar CD4^+^ T cell counts as compared to R VL/HIV at 3 months; however, it was significantly higher at 6–12 months. In the R VL/HIV group, the CD4^+^ T cell counts were significantly higher in patients who had not relapsed at 3 months, and at 6–12 months (Figures 7B and 7C). However, since blood was collected from only a single patient with P VL/HIV who relapsed during follow-up at 3 months and at 6–12 months, it is not possible to draw meaningful conclusions from these data.

CD8^+^ T cell counts were similar between both groups of patients with VL/HIV at all time points and were restored to levels similar to those of controls at EoT (Figure 7D, p > 0.05). No significant differences in CD8^+^ T cell counts were observed between patients with P and R VL/HIV who relapsed and those who did not relapse during follow-up (data not shown).

**Figure 8:**
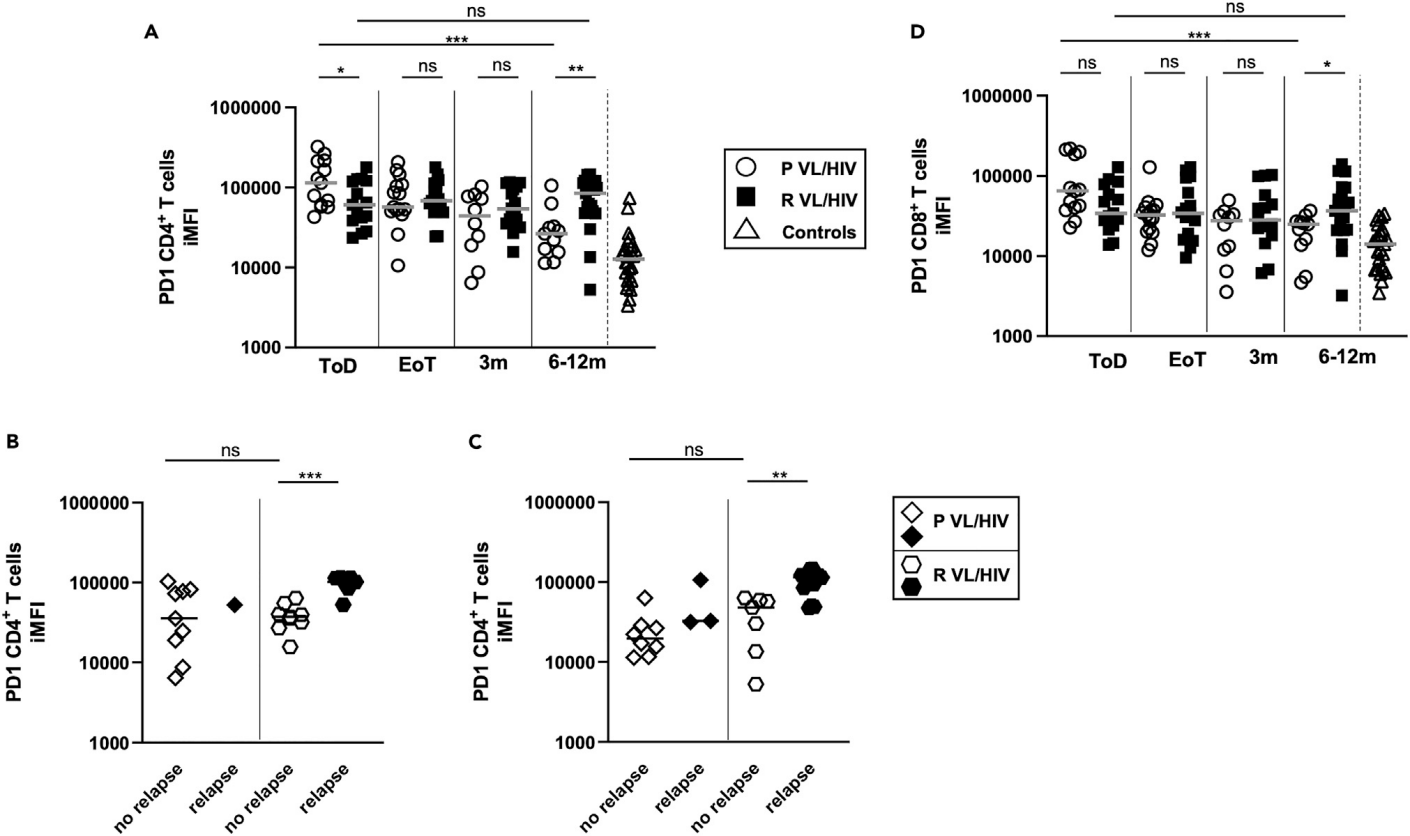
PD1 expression on CD4^+^ and CD8^+^ T cells (A) CD4 PD1 iMFI and (D). CD8 PD1 iMFI was measured by multiplying the % of T cells and the median fluorescence intensity of PD1 as measured by flow cytometry in the PBMCs of P VL/HIV (ToD: n = 13, EoT: n = 15, 3m: n = 10, 6-12m: n = 11), R VL/HIV patients (ToD: n = 15, EoT: n = 17, 3m: n = 16, 6-12m: n = 21) and healthy controls (n = 10). (B) Comparison of CD4 PD1 iMFI in patients with P VL/HIV who did not relapse (n = 9) and those who did relapse (n = 1) and patients with R VL/HIV who did not relapse (n = 9) and those who did relapse (n = 7) 3m after successful anti-leishmanial treatment. (C) Comparison of CD4 PD1 iMFI in patients with P VL/HIV who did not relapse (n = 8) and those who did relapse (n = 3) and patients with R VL/HIV who did not relapse (n = 7) and those who did relapse (n = 14) 6-12m after successful anti-leishmanial treatment. If a patient did not relapse during the 2 time points of follow-up and if a patient relapsed at both 3, 6–12 months, this is represented as 2 measurements. Statistical differences between two groups were determined using a Mann-Whitney test; statistical differences between the 4 different time points for each cohort of patients were determined by Kruskal-Wallis test.ToD = Time of Diagnosis; EoT = End of Treatment; 3m = 3 months post EoT; 6-12m = 6–12 months post EoT. ns = not significant.

We have previously shown that the expression level of PD1, an inhibitory receptor that can be associated with impaired effector functions, remained high on CD4^+^ T cells in patients with VL/HIV.^11^ Results presented in Figure 8A show that CD4 PD1 iMFI levels were higher throughout follow-up in both patients with P and R VL/HIV as compared to controls (p < 0.0001), and that CD4 PD1 iMFI levels decreased significantly in the P VL/HIV group, but not in the R VL/HIV group. There was no significant difference between the medians of CD4 PD1 iMFI in patients with P and R VL/HIV who did not relapse at both time points (Figures 8B and 8C). CD4 PD1 iMFI was significantly higher in patients with R VL/HIV who relapsed as compare to those who did not relapse (Figures 8B and 8C). However, since blood was collected from only a single patient with P VL/HIV who relapsed during follow-up at 3 months and three patients with P VL/HIV who relapsed at 6–12 months, it was not possible to draw meaningful conclusions from these data.

Although CD8 PD1 MFI remained significantly higher in both VL/HIV groups as compared to controls throughout follow-up (Figure 8D, p < 0.0001), CD8 PD1 iMFI decreased significantly over time in P VL/HIV, but not R VL/HIV. No significant differences in CD8 PD1 iMFI were observed between patients with P and R VL/HIV who relapsed and those who did not relapse during follow-up (data not shown).

### VL history is associated with increased relapse rate

Next, we tested the association between clinical and immunological factors that could be measured at time of diagnosis or at the end of treatment and the rate of VL relapse, reasoning that identifying associations at these early time points could help identify patients who could most benefit from additional intervention to prevent relapse. As shown in Table 2, prior history of VL relapse (recurrent VL) is an important risk factor for future relapse, with an estimated 1-year relapse rate of almost 79% (CI 49.7–91.9) in patients with recurrent VL compared to only 22.5% in patients experiencing their primary episode of VL (CI 0.3%–39.7%). In an effort to understand the causes of this further relapse, we tested the association of a wide range of clinical and immunological factors—measured at both time of diagnosis and end of treatment—with relapse rates and found only two other factors showing significant associations—CD4^+^ T cell count at end of treatment and parasite load measured by splenic aspirate at time of diagnosis. Neither of these factors remained significant in two-variable models that also included prior VL history, while VL history remained independently associated with relapse rate in these models.

**Table 2:** Type and duration of anti-leishmanial treatments

Number of P VL/HIV patients	Treatment	Duration of treatment (days)
1	SSG + PM	61
3	SSG	28
1	AmBisome	65
4	AmBisome	28
7	AmBisome + Miltefosine	28
1	AmBisome + Miltefosine	30
1	AmBisome + Miltefosine	40
1	AmBisome + Miltefosine	45
1	AmBisome + Miltefosine	62
1	AmBisome + pentamidine	30

Number of patients with R VL/HIV	Treatments	Duration of treatment (days)
1	SSG + PM	61
1	SSG	28
1	SSG	71
2	AmBisome	28
1	AmBisome	65
10	AmBisome + Miltefosine	28
1	AmBisome + Miltefosine	45
1	AmBisome + Miltefosine	62
1	AmBisome + Miltefosine	90
1	AmBisome + Miltefosine	52
1	AmBisome + Miltefosine	101
1	AmBisome + pentamidine	30
1	AmBisome + pentamidine	62
5		Treatment not completed

SSG = Sodium stibogluconate, PM = Paramomycin and AmBisome = Liposomal amphotericin B.

## Discussion

We and others have previously shown that patients with VL/HIV experience a high rate of relapse.^5^^,^^6^^,^^11^^,^^12^^,^^13^^,^^14^ Here, we analyzed this cohort of patients further by comparing clinical and immunological parameters in patients with VL/HIV who presented with their first episode of VL and those with a previous history of VL relapse.

Our results show that in agreement with previous studies,^5^^,^^6^ the relapse rate in patients with R VL/HIV is significantly higher. Our results also reveal that these patients have a shorter relapse-free survival. In addition, our results show a poorer recovery in weight gain and blood cell counts, higher spleen size, and parasite load in patients with R VL/HIV, as compared to patients with P VL/HIV:

- The median BMI of both groups of patients remained below 18.5 throughout the follow-up; however, the BMI increased significantly in the P, but not the R VL/HIV cohort. Malnutrition plays a crucial role in increased susceptibility to infection and/or disease severity by weakening both innate and acquired immunity.^29^^,^^30^ It is therefore possible that the lower BMI observed in patients with R VL/HIV at 6–12 months contributes to their poorer prognosis. Better management of malnutrition in both groups of patients with VL/HIV could improve their ability to mount an effective immune response.
- Splenomegaly increased in patients with R VL/HIV during the follow-up period, consistent with the observed increase in the parasite load over time in this group of patients.
- Although both groups of patients remained pancytopenic, the increase in WBC, RBC, and PLT counts following treatment was less efficient in patients with R VL/HIV. Bone marrow suppression can contribute to pancytopenia, and both VL and HIV infection have been associated with bone marrow failure.^31^^,^^32^^,^^33^ Both pathogens can infect hematopoietic stem/progenitor cells, and this can impair hematopoiesis. The higher parasite load observed in patients with R VL/HIV might contribute to the poorer recovery of all blood cell lineages.
- Although the parasite load decreased at EoT, the load as measured in blood remained higher from EoT onward in patients with R VL/HIV; at 3 and 6–12 months, it was similar to the load at ToD. These results are consistent with the higher relapse rate in these patients, 78.9% in the R VL/HIV versus 23.5% in the P VL/HIV cohort, indicating that patients with R VL/HIV have a poorer ability to control parasite replication.

To identify possible mechanisms responsible for this inability to efficiently control parasite replication, we analyzed immunological parameters. It has been shown that in splenic aspirates, IFNγ produced by CD4^+^ T cells contributes to parasite killing.^34^ We and others have speculated that in patients with VL/HIV, the impairment of antigen-specific IFNγ production by CD4^+^ T cells plays a key role in the inefficient control of parasite replication.^11^^,^^34^ Here, we show that the levels of antigen-specific IFNγ produced by whole blood cells were even further reduced in patients with R VL/HIV as compared to patients with P VL/HIV. This reduction in IFNγ might explain the higher parasite load detected in splenic aspirates and blood of patients with R VL/HIV. Antigen-specific IFNγ is mainly produced by CD4^+^ T cells in the whole blood assay.^34^ It is therefore plausible that low IFNγ levels are a consequence of low CD4^+^ T cell counts. Here, we show that both factors are likely to be involved: the CD4^+^ T cell counts are even lower in R VL/HIV and are accompanied with significantly lower production of antigen-specific IFNγ in the whole blood assay (WBA), as compared to P VL/HIV.

Low CD4^+^ T cell counts could be due to poor HIV control: in our study, many patients still had detectable viral loads throughout the follow-up despite being on ART. The recovery of CD4^+^ cells is often stunted in individuals who started ART with low CD4^+^ T cell counts^35^^,^^36^^,^^37^; but there is no ART regimen that has been shown to boost CD4^+^ T cell recovery. Inclusion of dolutegravir in first-line treatment could help to improve CD4^+^ T cell recovery through more efficient suppression of viral replication.^38^ It is also possible that there is some resistance to HIV drugs in this population: this has been reported to both first- and second-line treatments.^39^ Poor HIV control may also be due to difficulties accessing ART for the population of migrant workers during the agricultural season;^11^ indeed, this population is highly mobile and frequently lacks access to health facilities where they can get ART. Poor adherence to ART is also likely to play a role: whereas adherence counseling is available to patients with HIV, a recent study about ART adherence in the hospital in Gondar showed that adherence to ART was negatively associated with rural residence, lack of knowledge about HIV and ART, undisclosed HIV status to partners, and low CD4 count.^40^

T cell exhaustion is another factor that might account for the low antigen-specific IFNγ level observed in patients with VL/HIV. T cell exhaustion is characterized by a gradual loss of effector functions and co-expression of inhibitory receptors.^41^^,^^42^^,^^43^ PD1 is upregulated on T cells by signals such as IL-2, IL-7, type I IFNs, and signaling via the T cell receptor: it is therefore a marker of T cell activation.^44^ However, during chronic infection, the levels of PD1 remain high and are associated with T cell dysfunction.^43^ It is therefore plausible that in patients with VL/HIV, persistent antigenic stimulation due to *Leishmania* and HIV contributes to T cell exhaustion. T cells responding to chronic infection undergo progressive loss of functions; since patients with R VL/HIV have had a more intense and longer exposure to both pathogens, this could have resulted in higher PD1 CD4 iMFI and lower antigen-specific IFNγ production as compared to patients with P VL/HIV. Our results also show that whereas CD8^+^ T cell counts are restored at EoT, CD8 PD1 iMFI decreased significantly in patients with P VL/HIV but not in patients with R VL/HIV, suggesting that CD8^+^ T cells may also have an exhausted phenotype. It has been previously shown that CD8^+^ T cells from the blood of patients with VL have an anergic/exhausted phenotype, as shown by high levels of CTLA4 and PD1.^45^ However, we did not detect CTLA4 on T cells in patients with VL/HIV.^11^ In the study by Gautam et al., the authors show that CD8^+^ T cells contribute to the basal levels of IFNγ in whole blood, but not to the antigen-specific IFNγ production;^45^ similarly, the study by Kumar et al. showed that CD4^+^ T cells but not CD8^+^ T cells produce IFNγ in the WBA.^34^ We have previously discussed fundamental differences between patients with VL in India and in Ethiopia: whereas whole blood cells from patients with VL from Northwest Ethiopia have an impaired ability to produce antigen-specific IFNγ at ToD, this is not altered in patients with VL from India.^11^^,^^46^^,^^47^ We hypothesized that these differences were associated with the less severe VL symptoms in Indian patients as compared to patients from Northwest Ethiopia.^47^ In view of these differences, since we did not determine the phenotype of IFNγ-producing cells in our WBA, we cannot exclude the possibility that CD8^+^ T cells produce antigen-specific IFNγ. It is also possible that CD8^+^ T cells contribute to the elevated levels of IFNγ detected in the plasma of patients with VL/HIV.^11^

Patients with VL/HIV are likely to play a major role in the transmission of VL. A recent study by Singh et al. showed that patients with active VL, but not asymptomatic or successfully treated VL patients, can transmit the parasites to sand flies.^48^ Another study showed that VL/HIV co-infected individuals transmitted the parasites most efficiently to the sand fly vectors.^49^ Since patients with VL/HIV harbor higher parasite loads than patients with VL, these co-infected individuals are likely to be a significant reservoir for *L. donovani* and have a high potential for parasite transmission, thereby preventing the elimination of visceral leishmaniasis. From a public health perspective, it is important to note not only the high parasite burden in these patients but also the potential for drug resistance to emerge. Given the importance of parasite load to transmission, the contribution of treatment failure in patients with VL/HIV to the reservoir in the community needs to be determined and the cost to the health service as well as the health implications to the individual considered when determining management.

In summary, our results show that patients with VL/HIV who have a history of previous VL episodes relapse sooner and more often than those presenting with their first episode of VL. The poorer prognosis of R as compared to patients with P VL/HIV is accompanied by lower weight gain and recovery of WBC, RBC, and PLT counts; and lower production of antigen-specific IFNγ, lower CD4^+^ T cell counts, and higher CD4 and CD8 PD1 iMFI. In agreement with the study by Diro et al.,^5^ our results show that prior history of VL relapse is an important risk factor for future relapse. Furthermore, low CD4^+^ T cell counts at end of treatment and high parasite load at time of diagnosis are both associated with higher risk of VL relapse in survival models, but neither of these associations remained significant when adjusted for previous VL history, which remained independently associated with relapse risk. This suggests that neither of these factors are the sole drivers of continued relapse in patients with recurrent VL but emphasizes that a more complex immune dysfunction likely underlies the poor prognosis of recurrent VL.

Given the poor outcome predicted by VL/HIV co-infection and a history of relapse, specific measures should be tested to improve the long-term prognosis of these patients: an improvement of their ART treatment, such as inclusion of dolutegravir; more ART adherence counseling; a better follow-up of their nutritional status; and longer anti-leishmanial treatment. Additional molecular monitoring to inform the duration of anti-leishmanial treatment should be explored in future studies. Immune therapy, through PD1/PDL-1 blockade, that might improve the impaired production of antigen-specific IFNγ and/or through IFNγ administration^50^ could result in more efficient parasite killing in these patients. Such interventions might contribute to the prevention of further relapse.

### Limitation of the study

The main limitation of this study was the loss of follow-up. Since the majority of the study participants were migrant workers^11^ and were therefore traveling from farms to farms, the follow-up of this population was challenging.

Another limitation was that different anti-leishmanial treatments were used for different length of time (Table 3); furthermore, different ART treatments were also used (Table 4). The number of patients in each treatment group was therefore too small to test the impact of the treatments on VL relapses.

**Table 3:** ART

Number of P VL/HIV	ART at ToD	ART after EoT
18	TDF, 3 TC and EFV	
1	AZT, 3 TC and EFV	
2	Not on ART	TDF, 3 TC and EFV

**Number of R VL/HIV**		

25	TDF, 3 TC and EFV	
2	TDF 3 TC and ATV	
1	Not on ART	TDF, 3 TC and EFV

TDF: Tenofovir disoproxil fumarate, 3 TC: Lamivudine, EFV: Efavirenz, AZT: azidothymidine, ATV: atazanavir. ToD = Time of Diagnosis; EoT = End of Treatment.

**Table 4:** Survival analysis

			N	Time at risk (person-months)	Relapses (n)	1-year relapse rate (95% CI)^a^	Unadjusted hazard ratio (95% CI)^b^	Two-factor models Adjusted hazard ratio (95% CI)^c^
Total			49	324	19	0.51 (0.318,0.648)		
Prior VL history		Primary	28	198	4	0.225 (0.003,0.397)	1	1
		recurrent	21	126	15	0.789 (0.497,0.919)	**6.25 (2.05, 19.06)**	(**with parasite load)** **6.38 (0.85,48.7); p = 0.019**
								**With CD4 count EoT** **3.2534 (0.80, 13.3); p = 0.047**
Age		<33	23	171	9	0.5 (0.206,0.685)	1	
		≥ 33	26	153	10	0.511 (0.226,0.691)	1.20 (0.49, 2.96)	
Parasite load at ToD		<5	18	147	3	0.24 (0, 0.445)	1	1
		5 or 6	31	177	16	0.667 (0.413,0.811)	**4.56 (1.32,15.72)**	0.98 (0.10,9.35); p = 0.98
Previous relapses at ToD (in recurrent cases)		<3	16	78	7	0.7 (0.227, 0.884)	1	
		≥3	12	48	8	0.889 (0.295,0.983)	1.80 (0.63,5.10)	
WBC	ToD	<1600	22	147	8	0.482 (0.173, 0.676)	1	
		≥1600	27	177	11	0.532 (0.257, 0.706)	1.12 (0.45,2.79)	
	EoT	<2800	22	87	8	0.667 (0.258,0.850)	1	
		≥ 2800	27	237	11	0.438 (0.203,0.603)	0.49 (0.20,1.23)	
RBC	ToD	<3,100,000	23	171	8	0.44 (0.154,0.63)	1	
		≥ 3,100,000	26	153	11	0.579 (0.287,0.751)	1.61 (0.65,3.99)	
	EoT	<3,240,000	24	123	5	0.375 (0.05, 0.59)	1	
		≥ 3,240,000	25	201	14	0.58 (0.33, 0.74)	1.783 (0.6419,4.952)	
Platelet count	EoT	<79,000	24	144	8	0.457 (0.161, 0.648)	1	
		≥ 79,000	25	180	11	0.55 (0.269, 0.723)	1.13 (0.45, 2.81)	
	ToD	<207,000	24	105	8	0.583 (0.214, 0.779)	1	
		≥ 207,000	25	219	11	0.467 (0.22,0.635)	0.6693 (0.2688,1.667)	
Plasma HIV viral load	ToD	<255	19	90	8	0.571 (0.215, 0.766)	1	
		≥ 255	20	177	7	0.389 (0.117,0.577)	0.49 (0.18,1.38)	
	EoT	<150	14	81	7	0.583 (0.186,0.787)	1	
		≥ 150	19	165	9	0.483 (0.196,0.668)	0.67 (0.25, 1.81)	
CD4 count	ToD	<39.725	13	102	9	0.692 (0.305, 0.864)	1	
		≥ 39.725	13	117	4	0.375 (0, 0.611)	0.36 (0.11,1.18)	
	EoT	<115.5375	12	72	9	0.75 (0.333, 0.906)	1∗	1
		≥ 115.5375	12	126	3	0.287 (0,0.517)	**0.18**∗ **(0.05, 0.67)**	0.2644 (0.07, 1.07); p = 0.081
CD8 count	ToD	<146.85	12	108	4	0.5 (0,0.75)	1∗	
		≥ 146.85	12	81	2	0.25 (0,0.497)	0.57**∗** (0.10, 3.14)	
	EoT	<327.3375	12	105	3	0.325 (0,0.57)	1	
		≥ 327.3375	12	90	4	0.417 (0,0.67)	1.54 (0.34,6.89)	
CD4 PD1 iMFI	ToD	<69,069.6	13	87	4	0.44 (0.003,0.69)	1	
		≥ 69,069.6	14	120	5	0.44 (0.059, 0.672)	0.93 (0.25, 3.45)	
	EoT	<68,391	14	123	4	0.352 (0.002, 0.579)	1	
		≥ 68,391	15	117	7	0.518 (0.157, 0.724)	1.85 (0.54, 6.33)	
CD8 PD1 iMFI	ToD	<47,919.2	13	81	3	0.375 (0,0.635)	1	
		≥ 47,919.2	14	126	6	0.487 (0.112,0.704)	1.33 (0.33, 5.32)	
	EoT	<32,700	14	114	5	0.438 (0.057,0. 0.664)	1	
		≥ 32,700	15	126	6	0.444 (0.101,0.657)	1.09 (0.33,3.56)	
IFNγ	ToD	<0.189	18	120	10	0.625 (0.294,0.801)	1	
		≥ 0.189	19	129	4	0.342 (0,0.567)	0.36 (0.11, 1.15)	
	EoT	<2.058	15	117	11	0.733 (0.383,0.885)	1	
		≥ 2.058	16	120	5	0.375 (0.05, 0.59)	0.43 (0.15, 1.23)	
IL10	ToD	<0.765	18	108	9	0.67 (0.28,0.85)	1	
		≥ 0.765	19	141	5	0.34 (0.05,0.54)	0.42 (0.14, 1.27)	
	EoT	<1.14	15	132	8	0.533 (0.198, 0.728)	1	
		≥ 1.14	16	105	8	0.619 (0.213, 0.816)	1.24 (0.46, 3.31)	

Associations with relapse rate by prior VL history and strata of clinical and immunological measures at time of diagnosis and end of treatment. Bold entries in the unadjusted hazard ratio columns indicate two models for which a constant hazard ratio was rejected.

## STAR★Methods

### Key resources table

**Table:** 

REAGENT or RESOURCE	SOURCE	IDENTIFIER
**Antibodies**		

Anti-human CD4^FITC^	eBioscience	11-0049-80
Anti-human CD8^PE CY7^	eBioscience	25-0088-42
Anti-human PD1^PE^	eBioscience	12-9985-82
CD3/CD4/CD8a Antibody Cocktail, APC, FITC, PerCP-eFluor 710	eBioscience	22-0306-71

### Resource availability

#### Lead contact

Further information and requests for resources and reagents should be directed to and will be fulfilled by the lead contact, Pascale Kropf (p.kropf@imperial.ac.uk).

#### Materials availability

This study did not generate new unique reagents.

### Experimental model and subject details

For this cross-sectional study, we followed the cohort of 49 male VL/HIV patients (median age 33.5 ± 1.0 years) that was described in.^11^ Twenty-one patients presented with their first episode of VL (primary VL/HIV, P VL/HIV) and 28 with at least one previous episode of VL (recurrent VL/HIV, R VL/HIV, Table 1). The diagnosis of VL was based on positive serology (rK39) and the presence of *Leishmania* amastigotes in spleen or bone marrow aspirates.^51^ The diagnosis of HIV was done in accordance with the Ethiopian National HIV Screening Test Guidelines.^52^ Forty-six VL/HIV patients were on anti-retroviral therapy (ART) at the time of VL diagnosis; the remaining three started ART at the end of the anti-leishmanial treatment. All treatments were administered according to the Guideline for Diagnosis, Treatment and Prevention of Leishmaniasis in Ethiopia^53^ (Table 3). At the end of treatment, patients were discharged if they look improved, afebrile, had a smaller spleen size and an improved haematological profile (initial clinical cure). When there was no or insufficient clinical improvement, a tissue aspirate was performed to test for the presence of *Leishmania* amastigotes (Test Of Cure, TOC). If the TOC was still positive (i.e. incomplete cure), treatment was continued until TOC becomes negative or the patients have sufficient clinical improvement.^53^

The definitions of no relapse and relapse are defined as follows:

- no relapse: absence of clinical features of the disease 6 months after completion of the recommended dose and duration for VL treatment.
- Relapse: patient with VL treatment history presenting with clinical visceral leishmaniasis symptoms and is diagnosed with positive parasitology after successful completion of the treatment.

Antiretroviral therapy (ART) was provided according to the National Guidelines for Comprehensive HIV Prevention, Care and Treatment^52^^,^ Table 3).

Patients were recruited at four different time points:^11^ time of diagnosis (ToD); end of treatment; 3 months after the end of treatment (3m); and 6–12 months after the end of treatment (6-12m). We also recruited 10 non-endemic healthy controls (median age 30.0 ± 1.816 years).

This study was approved by the Institutional Review Board of the University of Gondar (IRB, reference O/V/P/RCS/05/1572/2017), the National Research Ethics Review Committee (NRERC, reference 310/130/2018) and Imperial College Research Ethics Committee (ICREC 17SM480). Informed written consent was obtained from each patient and control.

### Method details

#### Diagnosis and treatment of VL and HIV

The diagnosis of VL was based on positive for anti rK39 antibody by using IT Leish rapid test (Bio-Rad Laboratories, USA) and the presence of *Leishmania* amastigotes in spleen or bone marrow aspirate from the patients were smeared on a microscope slide, dried and fixed with absolute methanol for 2 min. The fixed slides were stained with Giemsa stain for 10 min and dried. Diagnosis was confirmed by identifying the amastigote stage of the parasite by microscopy using a 100X objective. The parasite load was graded by following a parasite grading system 6+: >100 parasites per field (under a 10X eyepiece and 100X oil-immersion lens), 5+: 10–100 parasites per field, 4+: 1–10 parasites per field, 3+: 1–10 parasites per 10 fields, 2+: 1–10 parasites per 100 fields, 1+: 1–10 parasites per 1000 fields, 0: 0 parasite per 1000 fields.^51^

- Diagnosis of HIV was done at the LRTC by detecting HIV-1 and HIV-2 antibodies. The testes were performed according to the Ethiopian national HIV screening test guideline, using a screening algorithm using STAT-PAK® (Chembio diagnostics systems. INC, USA), ABON (Abon Biopharm Co., Ltd. China), and SD BioLine (Standard Diagnostics. INC, India). According to the Ethiopian national HIV screening algorithm, STAT-PAK negative is reported as HIV negative. If STAT-PAK is positive, the sample will be tested with ABON and if ABON is positive the result will be reported as HIV positive. SD BioLine was used as a tiebreaker when there is a discrepancy between the results of STAT-PAK and ABON. This is, if STAT-PAK is positive, ABON is negative and SD BioLine is positive, the result will be reported as HIV positive. If STAT-PAK is positive, ABON negative, and SD BioLine negative the result will be reported as negative.

All ant leishmanial treatments dose and duration listed in (Table 2) were administered according to the Guideline for Diagnosis and Prevention of Leishmaniasis in Ethiopia.^53^ At EoT, all VL patients were clinically cured, defined as follows: patients look improved, are afebrile, usually have a smaller spleen size and have an improved haematological profile. A test of cure (TOC) was used for VL/HIV patients to decide if they could be discharged from hospital; a negative TOC is defined as follows: patients look improved, afebrile, and usually have a smaller spleen size, parasitological cure (absence of amastigotes in splenic aspirates) and an improved haematological profile.

The definitions for no relapse and relapse are defined by the by the “Guidelines for diagnosis, treatment and prevention of leishmaniasis in Ethiopia”^53^ as follows.

- no relapse: “*absence of clinical features of the disease 6 months after completion of the recommended dose and duration for VL*”.
- Relapse: “*patient with VL treatment history presenting with clinical visceral Leishmaniasis symptoms such as and is diagnosed with positive parasitology (LD bodies) after successful completion of the treatment*”.

##### HIV treatment

ART was provided according to the National guideline for comprehensive HIV prevention, care and treatment.^29^ 46 VL/HIV patients were on ART at the time of VL diagnosis, the remaining three started ART at the end of the anti-leishmanial treatment, all patients were on ART following the Ethiopian national guidelines for comprehensive HIV prevention, care, and treatment, and the following combination therapies were used Tenofovir disoproxil fumarate (TDF) + Lamivudine (3 TC) + Efavirenz (EFV), azidothymidine (AZT) + 3 TC + EFV or TDF + 3 TC + atazanavir (ATV).

#### Sample collection and processing

A total of 13 ml of blood was collected by venipuncture from patients and controls. The samples were divided as follows: 2.5 ml were added in PAXgene tubes for RNA-Seq, 8 ml in heparinised tubes; of these 3 ml were used for the WBA, 5 ml for flow cytometry; and 2.5 ml of blood were added into Ethylenediaminetetraacetic acid (EDTA) tubes for complete blood cell count, CD4^+^ and CD8^+^ T cell counts. The tubes were then centrifuged for 5 minutes at 3500 rpm and plasma was collected (from EDTA tubes) for the determination of HIV viral load and for the cytokine profile (from heparinised tubes).

- PBMCs were isolated from 5 ml of heparinised blood. 5 ml of blood was processed within 10 minutes after collection using density gradient centrifugation with Histopaque®-1077 (Sigma). 5 ml of Histopaque®-1077 was added into 15 ml centrifuge tubes and 5 ml of blood was layered at the top and centrifuged at 2200rpm for 30 minutes with the brake off. At the end of the centrifugation, the plasma at the top layer was separated and stored at −20°C for cytokine and chemokine measurement. PBMCs were collected, washed with phosphate buffer saline (PBS), and used for flow cytometry.^47^
- In the purified PBMCs the expression level of PD1 on CD4^+^ and CD8^+^ T cells was measured by using the following antibodies: CD4^FITC^, CD8^PE CY7^, and PD1^PE^ (eBioscience™). The data on PD1 expression are shown as the Median Fluorescent Intensity (MFI).
- CD4^+^ and CD8^+^ T cell counts were measured by using 100 μl of whole blood collected in EDTA. Cells were stained with CD4^FITC^, CD3 ^PerCP-eFluor® 710^, CD8α^APC^ monoclonal antibodies (eBioscience™) for 15 min at 4°C; red blood cells were lysed using BD FACS™ Lysing Solution for 5 min at room temperature. Acquisition was performed by using BD Accuri™ C6 flow cytometry (USA) and data were analysed using BD Accuri C6 analysis software version 1.0.264.21.
- Whole blood assay: soluble *leishmania* antigen (SLA) was prepared after growing *Leishmania* parasites from spleen or bone marrow aspirations, the following culture media was used; 500 ml of M199 medium (Sigma, USA) which was enriched with 25 mM hepes, 0.2 μM folic acid, 5 ml vitamin mix, 1 mM hemin, 1 mM adenine, 800 μM Biopterin, 5 ml of Penicillin streptomycin and 50 ml fetal bovine serum (Sigma, USA) to support the growth of *Leishmania* parasites.^47^ The culture medium was sterilised by filtration and stored at −20°C until it was used.

The parasites from spleen aspirates of 2 VL and 2 VL/HIV patients were cultured at room temperature and the cultures were monitored under the microscope until the parasites reached stationary phase. The parasites were pooled and transferred in a new multiple large volume up to 80 ml fresh medium and expanded to obtain high parasite concentration. Stationary-phase promastigotes were harvested and centrifuged at 4500 rpm for 20 minutes, the pellet was washed three times with cold PBS (Sigma, USA) and counted. The pellet was adjusted to 2 x 10^9^/ml and resuspended in SLA reagent (50 mM of EDTA, 50 mM of HCL, 100 mM of Phenylmethanesulfonyl fluoride (PMSF) (Sigma, USA), and 5 mg/ml of Leupeptin (Sigma, USA). The suspension was sonicated 4–5 times for 15 seconds at 10 Hz and centrifuged at 27,000xg for 30 minutes at 4°C. The lipid layer was removed from the surface of the supernatant. The remaining supernatant was ultra-centrifuged at 100,000xg for 4 hrs at 4°C with the breakoff. The supernatant was collected and the protein concentration was determined by using Pierce™ BCA Protein Assay Kit (Thermo Fisher Scientific) and absorbance was measured by using ELx800TM absorbance plate reader (BioTeK Instruments, USA). The SLA antigen was sterilized by filtration and the sterile SLA solution was stored at −20°C and used to stimulate whole blood (WB) cells in the whole blood assay.

Three millilitres of blood collected in heparinized tubes were used and 1 ml aliquots were distributed in 3 tubes and stimulated with SLA (10 μg/ml), Phytohaemagglutinin (PHA, Sigma) (10 μg/ml) and PBS as a negative control. The tubes were incubated for 24 hours at 37°C. The tubes were centrifuged at 3000rpm for 5 minutes and supernatants were collected and stored at −20°C to measure IFN-γ and IL10. IFNγ and IL-10 levels were measured in the supernatant of the WBA using IFN gamma Human ELISA Kit and IL-10 Human ELISA Kit (Invitrogen) according to the manufacturer’s instructions. The optical densities obtained with the unstimulated whole blood cells were subtracted from the optical densities obtained with PHA or SLA.^47^

- HIV viral load: plasma was isolated by centrifuging 2 ml of EDTA whole blood and frozen at −80°C. HIV viral load was measured in the Central Laboratory of the Amhara Public Health Institute, Bahir Dar, by using Abbott RealTime HIV-1 Qualitative (m2000sp), according to the manufacturer’s instructions.
- Cell count: white and red blood cell, and platelet counts were measured using a Sysmex XP-300T^M^ automated haematology analyser, (USA) following the manufacturer’s instruction.
- Relapse rate: Log-rank tests for duration of follow-up at event end points provided two-sided *p*-values; Kaplan-Meier curves are presented for visual interpretation. The primary outcome survival until 12 months of follow-up was completed; relapse was the only censoring event. Censoring events were reported at the pre-planned follow-up period (3 and 6–12 months) at which they were identified. Cox proportional-hazards regression analysis was used to estimate hazard ratios and 95% confidence intervals.
- mRNA: 2.5 ml of blood was collected in PAXgene blood RNA tubes, RNA extracted using the PAXgene 96 blood RNA kit (Qiagen) and Globin mRNA depleted using the GLOBINclear kit (Ambion). Sequencing libraries were prepared using the KAPA Stranded mRNA-Seq Kit (Roche) with 10 PCR cycles, then sequenced as 75bp paired-end reads on the Illumina HiSeq 4000 platform. Sequencing reads were mapped with Salmon v.1.30 against concatenated sequence of human gencode transcriptome release 34, transcripts for *L. donovani* LV9 from TriTrypDB release 46 Pseudo-counts were imported into R v4.0.3 using the tximport v1.18.0 and transformed into lengthscaled Transcripts Per Kilobase Million (TPM). Total *Leishmania* expression was quantified as the sum across all LV9 transcripts with the exception of feature LdLV9.27.2.206410, an 18S rRNA gene to which human transcripts also map.

### Quantification and statistical analysis

Data were evaluated for statistical differences as specified in the legend of each figure. The following tests were used: Mann-Whitney, Kruskal-Wallis or Spearman’s rank test. Differences were considered statistically significant at *p*<0.05. ∗=p<0.05, ∗∗=p<0.01, ∗∗∗=p<0.001 and ∗∗∗∗=p<0.0001. Unless otherwise specified, results are expressed as median ±SEM.

#### Survival analysis

One-year relapse rates were estimated using the Kaplan-Meier method, with quantitative factors split into two categories at the median value, with non-VL death and loss to follow-up as censoring events at EoT, 3-, 6- or 12-months follow-up. To formally test the significance of differences in relapse we used the Cox proportional-hazards model, initially estimating the hazard ratio with single-factor models for each covariate, where covariates were considered significantly associated with relapse if the 95% confidence interval for the hazard ratio did no overlap 1. We attempted to build a multivariable Cox model for the three significant factors, but this was not possible due to the level of missing data for end-of-treatment CD4 cell counts, so we instead built two-factor models for VL history and the two other significant factors (parasite load at time of diagnosis and CD4 count at end-of-treatment) to estimate the adjusted hazard ratio for each factor, testing their significance using ANOVA. These survival analyses used v3.2.7 of the survival package^54^ in R v4.0.2 (R Foundation for Statistical Computing, 2021 #3868).

## Data Availability

• All data reported in this paper will be shared by the lead contact upon reasonable request. • This paper does not report original code. • Any additional information required to reanalyse the data reported in this paper is available from the lead contact upon reasonable request.
