# Supplementary material for: Recurrent visceral leishmaniasis relapses in HIV co-infected patients are characterized by less efficient immune responses and higher parasite load
Source: iScience. 2022 Dec 23;26(2):105867. doi: 10.1016/j.isci.2022.105867 (PMC9845767; doi:10.1016/j.isci.2022.105867)
Supplement: Document S1. Tables S1–S5 [file mmc1.pdf]

iScience, Volume *26*

Supplemental information

Recurrent visceral leishmaniasis relapses in HIV co-infected patients are characterized by less efficient immune responses and higher parasite load

Yegnasew Takele, Tadele Mulaw, Emebet Adem, Rebecca Womersley, Myrsini Kaforou, Susanne Ursula Franssen, Michael Levin, Graham Philip Taylor, Ingrid Müller, James Anthony Cotton, and Pascale Kropf

Supplementary data
Table S1: *L. donovani* mRNAs, related to Figure 3

	**P VL/HIV**	**R VL/HIV**	***p* values**
**ToD**	136±608	1786±1229	0.1068
**EoT**	0.1±0.4	1.5±341.4	0.0007
**3m**	0.1±149.3	1047±1188	0.0099
**6-12m**	1.7±83.6	791±2847	0.0822
***p* values**	<0.0001	0.0056	

Quantification of the total expression of *L. donovani* mRNA in blood from P (ToD: n=12, EoT: n=16, 3m: n=8, 6-12m: n=7) and R VL/HIV patients (ToD: n=23, EoT: n=14, 3m: n=16, 6-12m: n=18)

Statistical differences between P and R VL/HIV patients at each time point were determined using a Mann-Whitney test; statistical differences between the 4 different time points for each cohort of patients were determined by Kruskal-Wallis test.

LD mRNA= *L. donovani* mRNA. P= Primary, R= recurrent; ToD=Time of Diagnosis; EoT=End of Treatment; 3m=3 months post EoT; 6-12m=6-12 months post EoT.

Table S2: Percentages of VL/HIV patients with detectable viral load and viral load, related to STAR Methods

% patients with detectable viral load	P VL/HIV	R VL/HIV	
ToD	66.7%	47.6%	
EoT	80.0%	44.4%	
3m	27.3%	50%	
6-12m	66.7%	38.1%	
**Viral load**	**P VL/HIV**	**R VL/HIV**	***p* values**
ToD	381±553679	200±581955	0.8452
EoT	150±50305	0.1±208331	0.4946
3m	542.5±160.9	2,873±99923	0.0799
6-12m	1,341±136928	629.5±199439	0.2758

Percentages of VL/HIV patients with detectable viral load: % of P VL/HIV (ToD: n=18, EoT: n=15, 3m: n=11, 6-12m: n=6) and R VL/HIV (ToD: n=21, EoT: n=18, 3m: n=16, 6-12m: n=6) patients who had detectable viral loads at ToD, EoT, 3 and 6-12m.

Viral load: HIV-1 viral load in plasma from P VL/HIV and R VL/HIV patients. Statistical difference between P and R VL/HIV was determined by Mann-Whitney test.

P= Primary, R= recurrent; ToD=Time of Diagnosis; EoT=End of Treatment; 3m=3 months post EoT; 6-12m=6-12 months post EoT.

Table S3: Liver size, related to STAR Methods

	**P VL/HIV**	**R VL/HIV**	***p* values**
**ToD**	4.0±0.6	2.0±0.7	0.2191
**EoT**	0.0±0.2	0.0±0.4	0.7136
**3m**	0.0±0.3	0.0±0.4	0.9263
**6-12m**	0.0±0.3	0.0±0.3	0.6133
***p* values**	<0.0001	0.009	

Liver size was measured in cm below the costal margin on P VL/HIV (ToD: n=21, EoT: n=17, 3m: n=14, 6-12m: n=18) and R VL/HIV (ToD: n=28, EoT: n=22, 3m: n=18, 6-12m: n=22) patients.

Statistical differences between P VL/HIV and R VL/HIV patients at each time point were determined using a Mann-Whitney test and statistical differences between the 4 different time points for each cohort of patients were determined by Kruskal-Wallis test.

P= Primary, R= recurrent; ToD=Time of Diagnosis; EoT=End of Treatment; 3m=3 months post EoT; 6-12m=6-12 months post EoT.

Table S4: White and red blood cell and platelet counts (cells/μl of blood), related to STAR Methods

**WBCs**	P VL/HIV	R VL/HIV	*p* values
ToD	1,500±165	1,900±374	0.0659
EoT	4,100±325	3,100±440	0.8399
3m	4,350±514	3,485±307	0.0606
6-12m	3,800±613	2,450±338	0.0388
p values	<0.0001	0.0162	
**RBCs**	P VL/HIV	R VL/HIV	*p* values
ToD	2,930,000±121,116	3,455,000±125,648	0.0090
EoT	3,235,000±133,465	3,490,000±172,403	0.1042
3m	4,460,000±191,972	3,760,000±153,258	0.0254
6-12m	4,200,000±243,966	3,750,000±176,013	0.1279
p values	<0.0001	0.7023	
**PLT**	P VL/HIV	R VL/HIV	*p* values
ToD	69,000±17,551	97,000±11,478	0.1291
EoT	207,500±29,018	172,000±19,964	0.6678
3m	222,000±29,542	118,500±11,618	<0.0001
6-12m	209,000±21,697	135,000±17,100	0.0246
p values	0.0018	0.0523	

White blood cell (WBC), red blood cell (RBC) and platelet (PLT) counts were measured in P VL/HIV (ToD: n=21, EoT: n=18, 3m: n=14, 6-12m: n=13) and R VL/HIV (ToD: n=28, EoT: n=21, 3m: n=18, 6-12m: n=14) patients.

Controls (n=25): WBC=6,700±439 cells/μl of blood; RBC=5,290,000±111,627 cells/μl of blood; PLT=269,000±11,705 cells/μl of blood.

Statistical differences between P VL/HIV and R VL/HIV patients at each time point were determined using a Mann-Whitney test and statistical differences between the 4 different time points for each cohort of patients were determined by Kruskal-Wallis test.

P= Primary, R= recurrent; ToD=Time of Diagnosis; EoT=End of Treatment; 3m=3 months post EoT; 6-12m=6-12 months post EoT.

Table S5: Production of IFNγ and IL-10 in response to PHA, related to Figure 6

IFNγ (pg/ml)	P VL/HIV	R VL/HIV	*p* values
**ToD**	10.5±12.0	5.7±114.4	0.7534
**EoT**	24.6±143.7	39.1±197.2	0.4746
**3m**	55.5±170.9	5.3±126.1	0.1476
**6-12m**	30.4±36.2	15.8±209.7	0.8002
***p* values**	0.3327	0.5585	
**IL-10 (pg/ml)**	**P VL/HIV**	**R VL/HIV**	***p* values**
**ToD**	17.1±13.1	30.0±28.5	0.6089
**EoT**	145.8 ±49.3	224.9±54.8	0.5298
**3m**	360.0±62.7	306.9±56.1	>0.9999
**6-12m**	486.7±147.0	131.8±43.2	0.0126
***p* values**	<0.0001	0.008	

Whole blood cells from P VL/HIV (ToD: n=16, EoT: n=16, 3m: n=9, 6-12m: n=6) and R VL/HIV (ToD: n=22, EoT: n=23, 3m: n=16, 6-12m: n=17) patients were cultured in the presence of d PHA. IFNγ and IL-10 levels in the supernatant were measured by ELISA after 24hrs.

Statistical differences between P and R VL/HIV patients at each time point were determined using a Mann-Whitney test; statistical differences between the 4 different time points for each cohort of patients were determined by Kruskal-Wallis test.

P= Primary, R= recurrent; ToD=Time of Diagnosis; EoT=End of Treatment; 3m=3 months post EoT; 6-12m=6-12 months post EoT.
